# Utility and limitations of Hepascore and transient elastography to detect advanced hepatic fibrosis in HFE hemochromatosis

**DOI:** 10.1038/s41598-021-94083-x

**Published:** 2021-07-19

**Authors:** Sim Yee Ong, Tiffany Khoo, Amanda J. Nicoll, Lyle Gurrin, Thomas Worland, Puraskar Pateria, Louise E. Ramm, Adam Testro, Gregory J. Anderson, Richard Skoien, Lawrie W. Powell, Grant A. Ramm, John K. Olynyk, Martin B. Delatycki

**Affiliations:** 1 Bruce Lefroy Centre, Murdoch Children’s Research Institute, Melbourne, VIC Australia; 2 The University of Melbourne, Melbourne, VIC Australia; 3 Department of Gastroenterology, Eastern Health, Box Hill, Victoria Australia; 4 Department of Gastroenterology, Fiona Stanley Hospital, Murdoch, WA Australia; 5 Monash University, Melbourne, VIC Australia; 6 Centre for Epidemiology and Biostatistics, Melbourne School of Population and Global Health, Carlton, VIC Australia; 7 Hepatic Fibrosis Group, QIMR Berghofer Medical Research Institute, Brisbane, QLD Australia; 8 Department of Gastroenterology, Austin Health, Heidelberg, VIC Australia; 9 Iron Metabolism Laboratory, QIMR Berghofer Medical Research Institute, Brisbane, QLD Australia; 10 Faculty of Medicine, The University of Queensland, Brisbane, Australia; 11 Department of Gastroenterology, Royal Brisbane and Women’s Hospital, Herston, QLD Australia; 12 School of Medical and Health Sciences, Edith Cowan University, Joondalup, WA Australia; 13 Victorian Clinical Genetics Services, Parkville, VIC Australia

**Keywords:** Liver diseases, Liver fibrosis

## Abstract

Aspartate aminotransferase-to-platelet ratio index (APRI) and Fibrosis-4 Index (Fib4) have been validated against liver biopsy for detecting advanced hepatic fibrosis in HFE hemochromatosis. We determined the diagnostic utility for advanced hepatic fibrosis of Hepascore and transient elastography compared with APRI and Fib4 in 134 newly diagnosed HFE hemochromatosis subjects with serum ferritin levels > 300 µg/L using area under the receiver operator characteristic curve (AUROC) analysis and APRI- (> 0.44) or Fib4- (> 1.1) cut-offs for AHF, or a combination of both. Compared with APRI, Hepascore demonstrated an AUROC for advanced fibrosis of 0.69 (95% CI 0.56–0.83; sensitivity = 69%, specificity = 65%; P = 0.01) at a cut-off of 0.22. Using a combination of APRI and Fib4, the AUROC for Hepascore for advanced fibrosis was 0.70 (95% CI 0.54–0.86, P = 0.02). Hepascore was not diagnostic for detection of advanced fibrosis using the Fib4 cut-off. Elastography was not diagnostic using either APRI or Fib4 cut-offs. Hepascore and elastography detected significantly fewer true positive or true negative cases of advanced fibrosis compared with APRI and Fib4, except in subjects with serum ferritin levels > 1000 µg/L. In comparison with APRI or Fib4, Hepascore or elastography may underdiagnose advanced fibrosis in HFE Hemochromatosis, except in individuals with serum ferritin levels > 1000 µg/L.

## Introduction

HFE hemochromatosis (HH) is an inherited iron overload disorder, most commonly due to homozygosity for the p.C282Y substitution in the HFE protein. The prevalence of homozygosity for this mutation approximates 1 in 190 in populations of northern European descent. It is now detected in the majority of individuals at early pre-clinical stages with readily available genetic and biochemical tests^1–6^. Early diagnosis and treatment normalises life expectancy and can prevent development of complications^7–9^. One of the main determinants of morbidity in HH is advanced hepatic fibrosis or cirrhosis (herein after termed advanced hepatic fibrosis, AHF)^7,8^. The term AHF encompasses Scheuer grades F3 and F4 fibrosis^10^. The ‘gold standard’ for assessing AHF has been through the use of liver biopsy. Currently, liver biopsy is recommended only in HH individuals with serum ferritin levels > 1000 µg/L and age greater than 40 years with abnormal liver function tests and/or hepatomegaly^7,11^, as it is not without complication^12,13^. For this reason, noninvasive approaches for diagnosis of AHF have gained traction^14–16^.

Some noninvasive fibrosis biomarkers have been validated against liver biopsy for detection of AHF in HH. The aspartate aminotransferase (AST)-to-platelet ratio index (APRI, cut-off value > 0.44) and Fibrosis-4 Index (Fib4, cut-off value > 1.1) demonstrate good diagnostic utility for the detection of AHF in HH with area under the receiver operator characteristic curve (AUROC) of 0.88 and 0.86, correctly identifying liver biopsy-diagnosed AHF in 85% and 80% of cases, respectively^17^. Another commonly used biomarker, Hepascore, is also available for detection of AHF in chronic liver diseases, but has not been validated in HH. Hepascore incorporates some of the markers of fibrogenesis and fibrinolysis to predict AHF^18^. It assesses clinical variables of sex and age and combines these with blood-based markers including bilirubin, gamma-glutamyl transferase (GGT), hyaluronic acid and alpha2-macroglobulin. A cut-off value for Hepascore > 0.50 has been suggested to be predictive of AHF in a range of different liver diseases other than HH^18,19^.

Transient elastography (TE) is an increasingly common non-invasive method fer detecting AHF in a range of liver diseases, but has not been validated in HH^20–27^. It relies on mechanical or acoustic modalities generating shear waves which are measured by ultrasound and converted to a stiffness estimate. It can be rapidly performed in an outpatient setting and provides immediate results. Limited studies have evaluated the performance of TE in subjects with HH^28,29^. Although liver biopsy was not performed in these studies, all subjects with TE > 8.7 kPa had evidence of AHF as determined noninvasively via the Fibrotest, Hepascore, Forns and Fib4 indices^28^. Since APRI and Fib4 have been recently validated as noninvasive biomarkers of advanced hepatic fibrosis in HH^17^, the aims of our study were to (1) determine the diagnostic utility of Hepascore and TE in comparison with APRI- and Fib4-determined cut-offs for the detection of probable AHF, and (2) evaluate their responses to phlebotomy treatment.

## Patients and methods

### Study participants

Newly diagnosed HH subjects were prospectively recruited between August 2012 to January 2018 across four different sites in Australia (Austin Health and Eastern Health in Victoria, the Royal Brisbane and Women’s Hospital, QIMR Berghofer Institute in Queensland, and Fiona Stanley Hospital in Western Australia) via referrals from medical practitioners, pathology companies which perform *HFE* genetic testing and through the Australian Red Cross LifeBlood Service. Inclusion criteria were homozygosity for HFE p.Cys282Tyr, age greater than 18 years and a serum ferritin level of more than 300 µg/L. Individuals were excluded if they had a history of significant alcohol intake (defined as > 60 g/day for males and > 40 g/day for females), a body mass index (BMI) of more than 35 kg/m^2^, were pregnant or had known liver disease from a cause other than HH. Patient demographics and clinical information were recorded. Liver biopsy was not a requirement for entry into the study^7,8^.

Blood was collected for measurement of serum ferritin level, transferrin saturation and parameters required for calculating the noninvasive biomarkers of fibrosis including platelets, ALT, AST, GGT, bilirubin, alpha-2-macroglobulin and hyaluronic acid. Noninvasive biomarker panel scores were calculated according to the following formulae:

- APRI: [(AST (U/L)/upper limit of normal) × 100/ platelet count (10^9^/L)]^16^
- Fib4: [age (years)  × AST (U/L)] / [platelet count (10^9^/L)  × ALT (U/L)^½^]^15^
- Hepascore: y/(1 + y), where y = exp [(-4.185818 – 0.0249 × age + 0.7464 × sex (male = 1, female = 0) + 1.0039 × α-2-macroglobulin (g/L) + 0.0302 × hyaluronic acid (µg/L) + 0.0691 × bilirubin (µmol/L) – 0.0012 × GGT (U/L)]^18^.

All subjects underwent TE by experienced and accredited operators using the Echosens FibroScan device as per the manufacturer’s recommendations. Transducer placement was at 9/10 or 10/11 rib spaces. A reliable data set was defined as an interquartile range/median value ratio × 100 equalling less than 30%.

We evaluated two noninvasive biomarker models for the detection of AHF in HH (models 1 and 2):

**Model 1** used individual values for APRI > 0.44 or Fib4 > 1.1 to define the presence of probable noninvasive biomarker-detected AHF (nAHF). Absence of nAHF was defined by APRI ≤ 0.44 or Fib4 ≤ 1.1 (termed ‘no nAHF’). The Hepascore and TE measurements were compared against APRI or Fib4 in the nAHF and ‘no nAHF’ groups.

**Model 2** was based on the combination of APRI > 0.44 and Fib4 > 1.1 to define the presence of nAHF, similar to the method used by Papadopoulos et al.^30^. Based on the data of Chin et al.^17^ 67% of all HH subjects who had both APRI > 0.44 and Fib4 > 1.1 had AHF confirmed by liver biopsy. Of those who had both APRI ≤ 0.44 and Fib4 ≤ 1.1, 97% did not have AHF. For model 2, nAHF was defined as being present when subjects had both an APRI > 0.44 and a Fib4 > 1.1. Absence of nAHF was defined as both APRI ≤ 0.44 and Fib4 ≤ 1.1 in the same subject (no nAHF). Subjects with an elevation in only one of either APRI or Fib4 values (i.e. above the respective nAHF cut-offs) were classified as ‘indeterminate’. The Hepascore and TE values were compared in the nAHF and ‘no nAHF’ groups.

All subjects provided informed consent. Human Research Ethics approvals were obtained from Austin Health (HREC/15/Austin/56 and HREC/12/Austin/19), Eastern Health (LR81/2015), Queensland Metro North Hospital and Health Service (HREC/15/QRBW/502) and the Western Australian South Metropolitan Health Service (15-147).

### Statistical analysis

Data are presented as the mean ± SE, unless otherwise specified. Comparisons between continuous variables were performed using analysis of variance or t-test (unpaired or paired) whilst categorical analysis was conducted using chi-square analysis. AUROC curve analysis was performed for evaluation of diagnostic performance, sensitivity and specificity of Hepascore and TE in comparison with either APRI or Fib4 (Model 1) or the combination of APRI and Fib4 (Model 2) (Prism 9.0, GraphPad Software). Cut-off values for Hepascore or TE were derived for the maximal combination of sensitivity and specificity(Youden’s index) in each AUROC analysis. Statistical significance was defined as P < 0.05.

### Data transparency statement

No data, analytical methods or study materials will be made available to other researchers.

### Statement on guidelines

This study is in compliance with the Australian Code for the Responsible Conduct of Research, 2018.

## Results

A total of 150 individuals were recruited to the study. There were incomplete data for 16 individuals, leaving a cohort of 134 people. The population was predominantly male (68%) with a mean age of 44 years. The mean BMI was 26.5 kg/m^2^. Liver biochemistry was within accepted reference ranges, as were the platelet count and international normalised ratio (INR) (Table 1). The mean serum ferritin level of the cohort was 691 µg/L with 20 subjects having a serum ferritin level > 1000 µg/L.

**Table 1:** Baseline demographic and biochemical characteristics of study patients.

Gender (n, male, female)	91, 43
Age (years)	44.4 ± 1.3
BMI (kg/m^2^)	26.5 ± 0.4
**Biochemistry**	
Serum ferritin (µg/L)	691 ± 43
Serum ferritin > 1000 µg/L (n)	20
Transferrin saturation (%)	66 ± 1.6
Platelets (×10^9^/L)	236 ± 4
ALT (U/L)	35 ± 2
AST (U/L)	26 ± 1
GGT (U/L)	27 ± 2
INR	1.0 ± 0
**Noninvasive fibrosis biomarkers**	
TE (kPa)	5.1 ± 0.2
Hepascore	0.23 ± 0.01
APRI	0.33 ± 0.01
Fib4	0.92 ± 0.04

All data are presented as the mean ± SE.

ALT: alanine aminotransferase; APRI: aspartate aminotransferase to platelet ratio index; AST: aspartate aminotransferase; BMI: body mass index; Fib4: fibrosis-4; GGT: gamma-glutamyl transferase; INR: international normalised ratio; TE: transient elastography.

### Model 1—performance of Hepascore and TE in comparison with either APRI or Fib4

**Figure 1:**
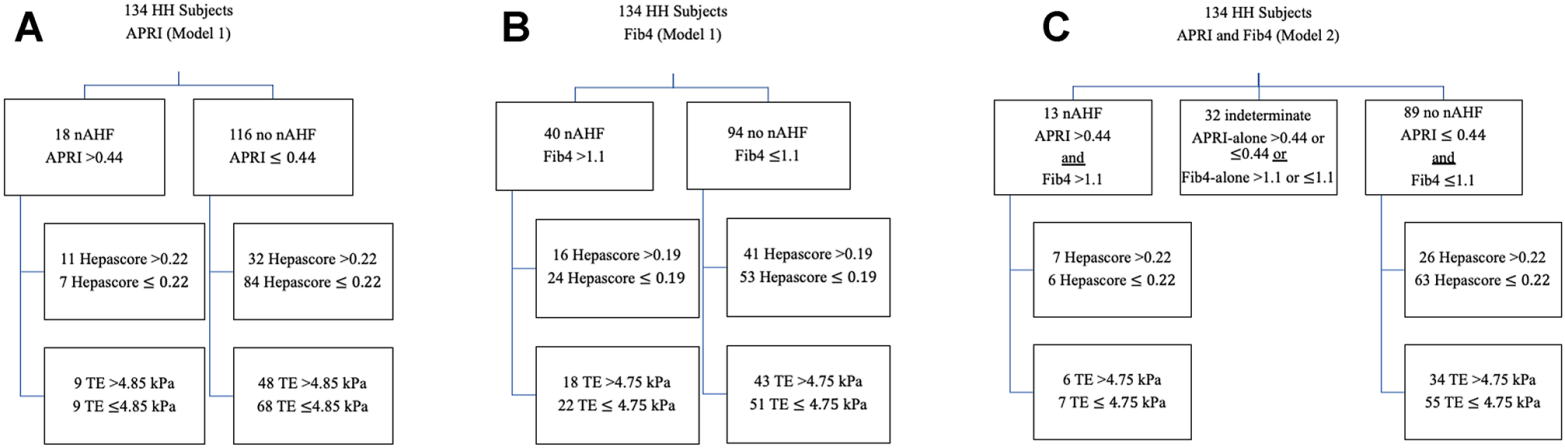
(**A**) Study subjects classified as having noninvasive biomarker detected advanced hepatic fibrosis (nAHF) on the basis of APRI > 0.44 (Model 1). Subjects with APRI ≤ 0.44 were classified as not having nAHF (no nAHF). The numbers of subjects in each of these categories detected by Hepascore (cut-off 0.22) or transient elastrography (TE, cut-off 4.85 kPa) are shown in each box. (**B**) Study subjects classified as having noninvasive biomarker detected advanced hepatic fibrosis (nAHF) on the basis of Fib4 > 1.1 (Model 1). Subjects with Fib4 ≤ 1.1 were classified as not having nAHF (no nAHF). The numbers of subjects in each of these categories detected by Hepascore (cut-off 0.19) or transient elastrography (TE, cut-off 4.75 kPa) are shown in each box. (**C**) Study subjects classified as having noninvasive biomarker detected advanced hepatic fibrosis (nAHF) on the basis of APRI > 0.44 and Fib4 > 1.1. Subjects with APRI ≤ 0.44 and Fib4 ≤ 1.1 were classified as not having nAHF (no nAHF). Those subjects not meeting any of the preceding criteria were classified as indeterminate. The numbers of subjects in each of the nAHF or no nAHF categories detected by Hepascore (cut-off 0.22) or transient elastrography (TE, cut-off 4.75 kPa) are shown in each box.

**Figure 2:**
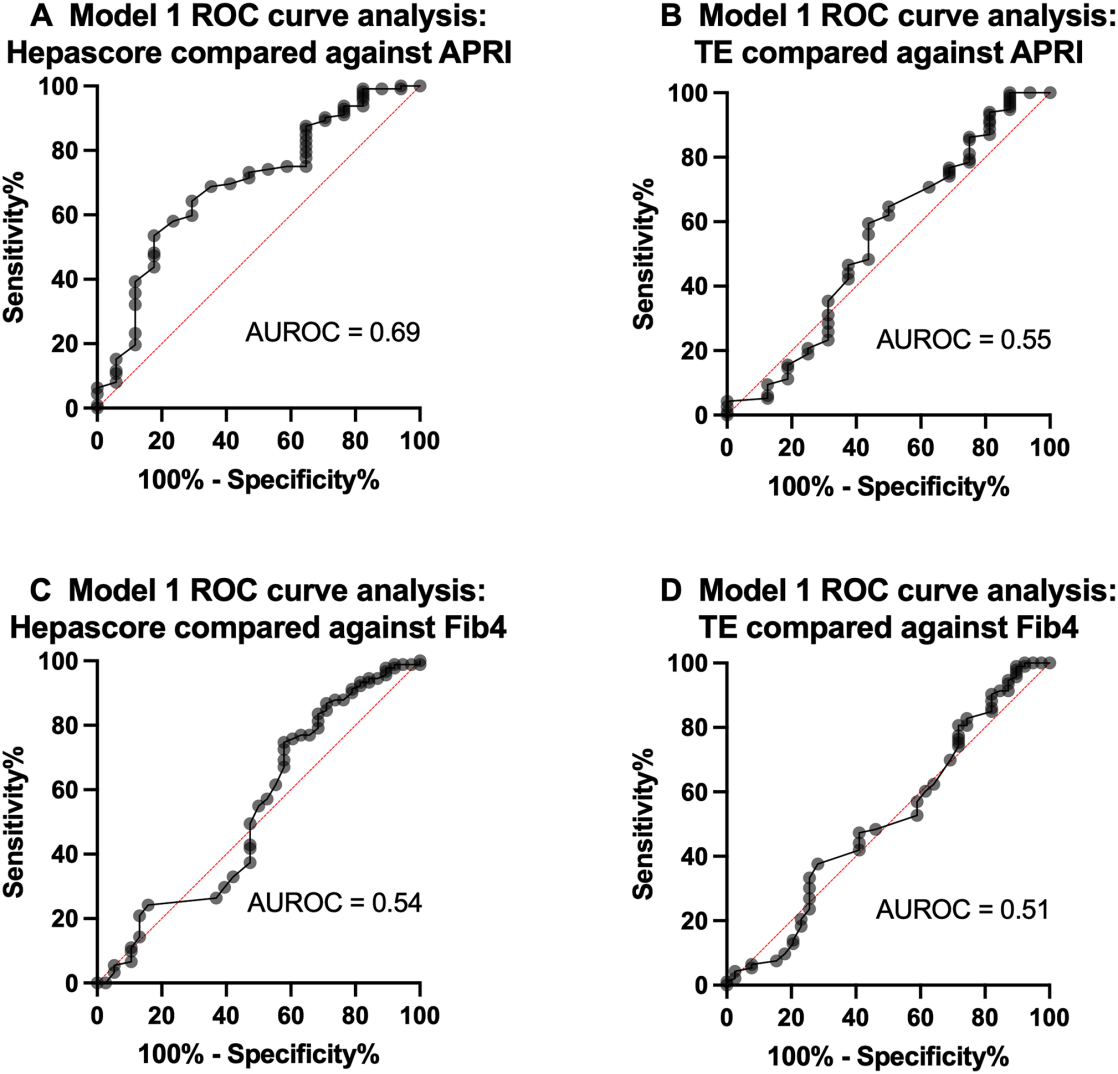
Receiver-operator characteristic curve analysis for Model 1 comparing Hepascore against APRI > 0.44 (**A**), TE against APRI > 0.44 (**B**), Hepascore against Fib4 > 1.1 (**C**), or TE against Fib4 > 1.1 (**D**).

Eighteen of 134 subjects (13%) had an APRI > 0.44 (nAHF) whilst 116 had an APRI ≤ 0.44 (87%, no nAHF) (Fig. 1A). The AUROC curve analysis for the diagnosis of nAHF based on elevated APRI demonstrated that Hepascore had an AUROC of 0.69 (95% CI 0.56–0.83, P = 0.01) and a maximum sensitivity and specificity of 69% and 65%, respectively, at a cut-off value of 0.22 (Fig. 2A). TE had an AUROC of 0.55 (95% CI 0.38–0.71, P = 0.56), and a maximum sensitivity and specificity of 56% and 56%, respectively, at a cut-off value of 4.85 kPa (Fig. 2B). Of the 18 subjects with APRI values consistent with nAHF, 11 were detected with Hepascore > 0.22 (61% true positive, 39% false negative, P < 0.01, Chi-square) and 9 with TE > 4.85 kPa (50% true positive, 50% false negative, P < 0.001, Chi-square). Of the 116 subjects with APRI values ≤ 0.44, 84 were detected with Hepascore ≤ 0.22 (72% true negative, 28% false positive, P < 0.001, Chi-square) and 68 with TE ≤ 4.85 kPa (59% true negative, 41% false positive, P < 0.001, Chi-square). Subjects with nAHF classified using APRI had significantly higher serum ferritin, ALT, AST and Hepascore levels compared with those who did not have nAHF (Table 2). Six of 18 subjects with APRI-determined nAHF had serum ferritin values > 1000 µg/L; all were detected using Hepascore > 0.22 or TE > 4.85 kPa.

**Table 2:** Characteristics of study subjects in Model 1 (APRI or Fib4) and Model 2 (combined APRI and Fib4 scores).

	nAHF	No nAHF
**Model 1**		
APRI		
Gender (n, male, female)	12, 6	78, 38
Age (years)	48 ± 4	44 ± 1
Ferritin (µg/L)	1118 ± 191	625 ± 37***
Transferrin saturation (%)	74 ± 4	65 ± 2
ALT (U/L)	73 ± 11	29 ± 1***
AST (U/L)	46 ± 5	23 ± 0.7***
Hepascore	0.35 ± 0.06	0.21 ± 0.01**
TE (kPa)	5.9 ± 0.9	4.9 ± 0.13
Fib4		
Gender (n, male, female)	27, 13	64, 30
Age (years)	57 ± 1.6	39 ± 1***
Ferritin (µg/L)	875 ± 114	613 ± 35*
Transferrin saturation (%)	67 ± 3	65 ± 2
ALT (U/L)	37 ± 3	34 ± 3
AST (U/L)	31 ± 1	25 ± 1*
Hepascore	0.27 ± 0.04	0.22 ± 0.01
TE (kPa)	5.4 ± 0.4	4.9 ± 0.1
**Model 2**		
Gender (n, male, female)	8, 5	60, 29
Age (years)	56 ± 3	40 ± 1***
Ferritin (µg/L)	1188 ± 250	595 ± 34***
Transferrin saturation (%)	74 ± 4	65 ± 2
ALT (U/L)	54 ± 6	29 ± 1***
AST (U/L)	38 ± 3	22 ± 1***
Hepascore	0.17 ± 0.11	0.05 ± 0.02
TE (kPa)	6.0 ± 1.1	4.9 ± 0.1

All data are presented as the mean ± SE.

ALT: alanine aminotransferase; APRI: aspartate aminotransferase to platelet ratio index; AST: aspartate aminotransferase; Fib4: fibrosis-4; GGT: gamma-glutamyl transferase; nAHF: noninvasive biomarker advanced hepatic fibrosis; No nAHF: no noninvasive biomarker advanced hepatic fibrosis; TE: transient elastography.

*P < 0.05, **P < 0.01, ***P < 0.0001 compared with nAHF group (unpaired t test).

Forty of 134 subjects (30%) had a Fib4 > 1.1 (nAHF) whilst 94 had a Fib4 ≤ 1.1 (70%, no nAHF) (Fig. 1B). The AUROC curve analysis for the diagnosis of nAHF based on elevated Fib4-alone demonstrated that Hepascore had an AUROC of 0.54 (95% CI 0.41–0.65, P = 0.52), and a maximum sensitivity and specificity of 49% and 53%, respectively, at a cut-off value of 0.19 (Fig. 2C). TE had an AUROC of 0.51 (95% CI 0.40–0.63, P = 0.80), and a maximum sensitivity and specificity of 48% and 54%, respectively, at a cut-off value of 4.75 kPa (Fig. 2D). Of the 40 subjects with Fib4 values consistent with nAHF, 16 were detected with Hepascore > 0.19 (40% true positive, 60% false negative, P < 0.001, Chi-square) and 18 with TE > 4.75 kPa (45% true positive, 55% false negative, P < 0.001, Chi-square). Of the 94 subjects with Fib4 values ≤ 1.1, 53 were detected with Hepascore ≤ 0.19 (56% true negative, 44% false positive, P < 0.001, Chi-square) and 51 with TE ≤ 4.75 kPa (54% true negative, 46% false positive, P < 0.001, Chi-square). Subjects with nAHF classified using Fib4 had significantly higher age, serum ferritin, and AST levels compared with those who did not have nAHF (Table 2). Eight of 40 subjects with Fib4-determined nAHF had serum ferritin values > 1000 µg/L; 6 of these were detected with Hepascore > 0.19 and 5 with TE > 4.75 kPa.

Overall for Model 1, Hepascore demonstrated limited diagnostic utility using a cut-off value of 0.22 for detecting AHF in comparison with APRI. Hepascore was not diagnostically useful for detection of AHF in comparison with Fib4. TE was not diagnostic in comparison with either APRI or Fib4. Both Hepascore and TE detected significantly lower numbers of true positive or true negative cases compared with APRI or Fib4, except in subjects with serum ferritin levels > 1000 µg/L.

### Model 2—performance of Hepascore and TE in comparison with combined APRI and Fib4

**Figure 3:**
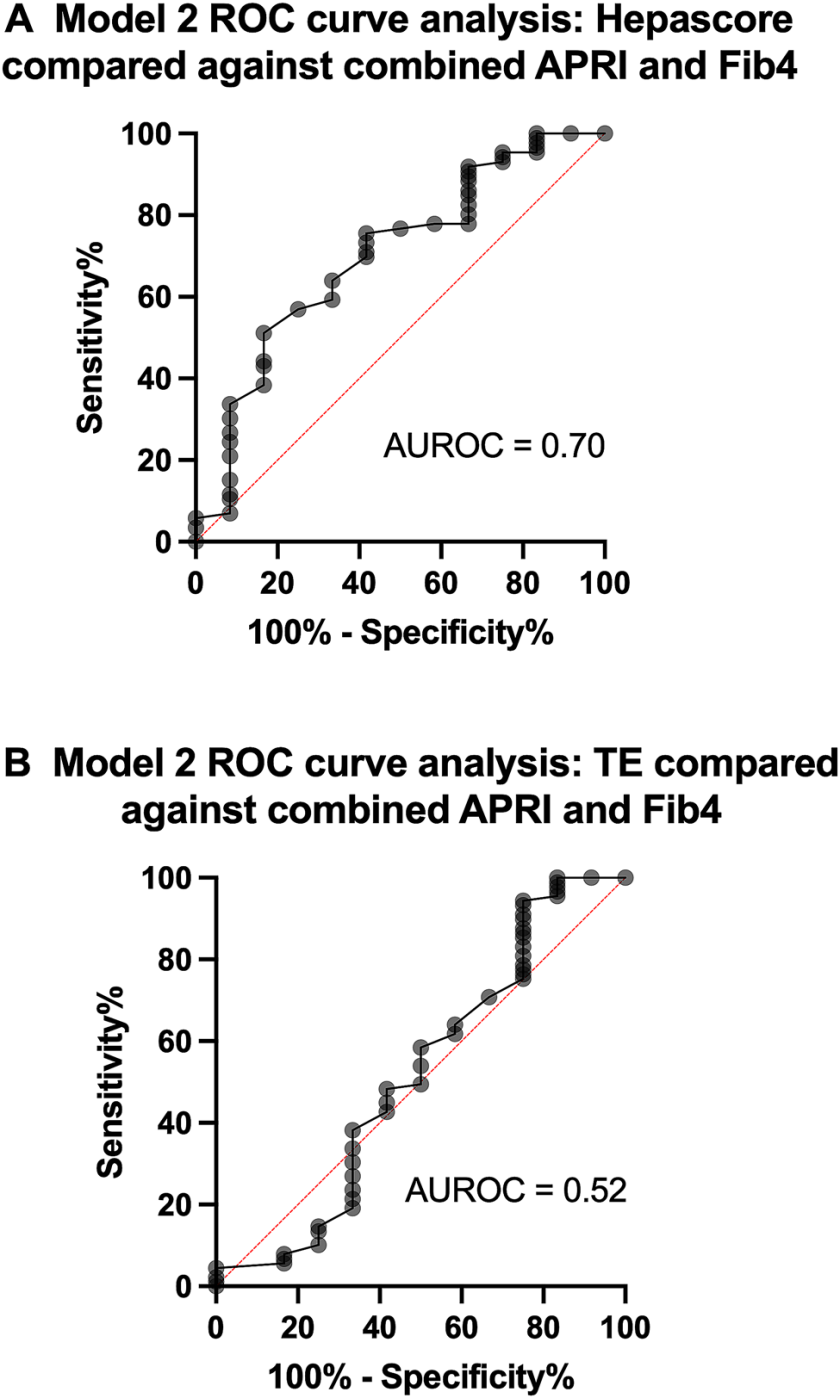
Receiver-operator characteristic curve analysis for Model 2 comparing either Hepascore (**A**) or TE (**B**) against the combination of APRI > 0.44 and Fib4 > 1.1.

Thirteen of 134 subjects (10%) had both an elevated APRI and Fib4 and were defined by this model as having nAHF (Fig. 1C). Eighty-nine subjects (66%) had APRI and Fib4 values below the cut-off levels and were defined as not having nAHF (no nAHF), whilst 32 (24%) were indeterminate. The AUROC curve analysis for the diagnosis of nAHF demonstrated that Hepascore had an AUROC of 0.70 (95% CI 0.54–0.86, P = 0.02), and a maximum sensitivity and specificity of 64% and 67%, respectively, at a cut-off value of 0.22 (Fig. 3A). TE had an AUROC of 0.52 (95% CI 0.31–0.72, P = 0.85), and a maximum sensitivity and specificity of 49% and 50%, respectively, at a cut-off value of 4.75 kPa (Fig. 3B). Seven of 13 nAHF subjects (54% true positive, 46% false negative) were detected using Hepascore > 0.22 (P < 0.01, Chi-square) whilst 6 of 13 nAHF subjects (46% true positive, 54% false negative) were detected using TE > 4.75 kPa (Fig. 1C, P < 0.01, Chi-square). Of the 89 subjects who did not have nAHF using this model (no nAHF), Hepascore and TE correctly classified 63 (71% true negative, 29% false positive, P < 0.001, Chi-square) and 55 (62% true negative, 38% false positive, P < 0.001, Chi-square), respectively. Subjects with nAHF classified using Model 2 had significantly higher age, serum ferritin, ALT and AST levels compared with those who did not (Table 2). Five of 13 subjects with nAHF had serum ferritin values > 1000 µg/L, 4 of these were identified with Hepascore > 0.22 and TE > 4.75 kPa.

Overall for Model 2, Hepascore demonstrated some diagnostic utility for detecting AHF, but detected significantly lower numbers of true positive and true negative cases compared with APRI and Fib4 combined. TE was not diagnostic. Both Hepascore and TE correctly identified likely AHF in most subjects with serum ferritin levels > 1000 µg/L.

### Response of noninvasive fibrosis markers to phlebotomy treatment

For those subjects classified as having nAHF on the basis of APRI > 0.44, there were significant reductions in APRI and serum ferritin levels with treatment (Table 3). There were no treatment-related effects on Fib4, TE or Hepascore. Serum ferritin levels declined significantly with treatment in those subjects classified as not having nAHF on the basis of APRI ≤ 0.44 in Model 1. However, there were no treatment-related effects on APRI, Fib4, TE or Hepascore.

**Table 3:** Comparison of noninvasive biomarkers pre- and post-treatment in the subjects with either noninvasive biomarker-detected advanced hepatic fibrosis (nAHF) using APRI > 0.44 or no evidence of noninvasive biomarker-detected advanced hepatic fibrosis (no nAHF) using APRI ≤ 0.44 in Model 1.

	APRI	Fib4	TE	Hepascore	Ferritin (µg/L)
**nAHF**					
Pre-treatment	0.64 ± 0.05	1.4 ± 0.2	5.9 ± 0.9	0.35 ± 0.06	1118 ± 191
Post-treatment	0.44 ± 0.05*	1.3 ± 0.2	4.5 ± 0.5	0.30 ± 0.08	317 ± 37**
**No nAHF**					
Pre-treatment	0.28 ± 0.01	0.84 ± 0.03	4.9 ± 0.1	0.21 ± 0.01	624 ± 37
Post-treatment	0.30 ± 0.03	0.98 ± 0.13	5.0 ± 0.2	0.20 ± 0.02	338 ± 21***

Results are shown as mean ± SE.

APRI: aspartate aminotransferase to platelet ratio index; Fib4: fibrosis-4; nAHF: noninvasive biomarker advanced hepatic fibrosis; No nAHF: no noninvasive biomarker advanced hepatic fibrosis; TE: transient elastography.

*P < 0.05, **P < 0.01, ***P < 0.0001 compared with pre-treatment (paired t test).

For those subjects classified as having nAHF on the basis of Fib4 > 1.1, there were significant reductions in APRI, Fib4 and serum ferritin levels with treatment (Table 4). There were no treatment-related effects on TE or Hepascore. Serum ferritin levels declined significantly with treatment in those subjects classified as not having nAHF on the basis of Fib4 ≤ 1.1 in Model 1. However, there were no treatment-related effects on APRI, Fib4, TE or Hepascore.

**Table 4:** Comparison of noninvasive biomarkers pre- and post-treatment in the subjects with either noninvasive biomarker-detected advanced hepatic fibrosis (nAHF) using Fib4 > 1.1 or no evidence of noninvasive biomarker-detected advanced hepatic fibrosis (no nAHF) using Fib4 ≤ 1.1 in Model 1.

	APRI	Fib4	TE	Hepascore	Ferritin (µg/L)
**nAHF**					
Pre-treatment	0.41 ± 0.02	1.5 ± 0.07	5.4 ± 0.4	0.27 ± 0.04	875 ± 114
Post-treatment	0.34 ± 0.03**	1.3 ± 0.1*	4.9 ± 0.3	0.25 ± 0.04	377 ± 41***
**No nAHF**					
Pre-treatment	0.29 ± 0.02	0.68 ± 0.02	4.9 ± 0.1	0.22 ± 0.02	613 ± 35
Post-treatment	0.30 ± 0.04	0.89 ± 0.16	4.9 ± 0.2	0.20 ± 0.02	319 ± 21***

Results are shown as mean ± SE.

APRI: aspartate aminotransferase to platelet ratio index; Fib4: fibrosis-4; nAHF: noninvasive biomarker advanced hepatic fibrosis; No nAHF: no noninvasive biomarker advanced hepatic fibrosis; TE: transient elastography.

*P < 0.05, **P < 0.01, ***P < 0.0001 compared with pre-treatment (paired t test).

In Model 2, there were treatment-related significant reductions in serum ferritin levels in those with or without nAHF (Table 5). However, there were no treatment-related effects in the two fibrosis groups with regards to APRI, Fib4, Hepascore or TE.

**Table 5:** Comparison of noninvasive biomarkers pre- and post-treatment in the subjects with either noninvasive biomarker-detected advanced hepatic fibrosis (nAHF) or no evidence of noninvasive biomarker-detected advanced hepatic fibrosis (no nAHF) using Model 2.

	APRI	Fib4	TE	Hepascore	Ferritin (µg/L)
**nAHF**					
Pre-treatment	0.77 ± 0.12	1.6 ± 0.3	6.0 ± 1.1	0.17 ± 0.06	1188 ± 250
Post-treatment	0.57 ± 0.20	1.4 ± 0.3	4.4 ± 0.7	0.29 ± 0.18	328 ± 53*
**No nAHF**					
Pre-treatment	0.25 ± 0.01	0.68 ± 0.03	4.8 ± 0.2	0.20 ± 0.01	484 ± 19
Post-treatment	0.30 ± 0.04	0.88 ± 0.16	4.9 ± 0.2	0.19 ± 0.01	321 ± 22***

Results are shown as mean ± SE.

APRI: aspartate aminotransferase to platelet ratio index; Fib4: fibrosis-4; nAHF: noninvasive biomarker advanced hepatic fibrosis; No nAHF: no noninvasive biomarker advanced hepatic fibrosis; TE: transient elastography.

*P < 0.05, ***P < 0.0001 compared with pre-treatment (paired t test).

## Discussion

Early diagnosis of AHF in HH is important for guiding clinical management. Since most subjects with HH are now detected prior to the development of clinical sequelae of iron overload, there is a need to define useful noninvasive methods for assessment of AHF to ensure that only those at highest risk of AHF progress to liver biopsy. This type of approach is occurring across a broad spectrum of liver diseases previously dependent on liver biopsy for accurate fibrosis staging^24^. What is clearly apparent is the variation in cut-off thresholds and suitability of these methods in various different liver diseases^24^. Furthermore, liver biopsy is not well suited for routine follow-up of fibrosis following treatment due to its invasive nature and associated risks^8,9^. In HH, APRI and Fib4 were recently shown to be accurate for detection of liver biopsy-diagnosed AHF with cut-off levels greater than 0.44 and 1.1, respectively^17^. APRI was also useful in monitoring fibrosis regression following treatment. These cut-off values in HH were substantially lower than those reported for other liver disease aetiologies^14,15,24^. With this knowledge, we designed the current real-world study of community-dwelling subjects with HH to determine the utility of two other commonly available noninvasive methods of detecting hepatic fibrosis, Hepascore and TE, using APRI- and Fib4-determined cut-offs as surrogates for AHF.

We compared Hepascore and TE with either APRI or Fib4 (model 1) or a combination of APRI and Fib4 (model 2). Hepascore demonstrated only fair diagnostic utility for the diagnosis of nAHF based on elevated APRI (AUROC 0.69, 95% CI 0.56–0.83, P = 0.01) or the combination of APRI and Fib4 (AUROC 0.70, 95% CI 0.54–0.86, P = 0.02). Nonsignificant diagnostic utility was demonstrated for Hepascore in comparison with Fib4 (AUROC 0.54, 95% CI 0.41–0.65, P = 0.52). Similarly, TE demonstrated no diagnostic utility for AHF in comparison with APRI, Fib4 or the combination of APRI and Fib4. Hepascore (using a cut-off value of 0.22) and TE (using a cut-off value of 4.75 kPa) detected significantly less true positive or true negative cases of AHF compared with APRI or Fib4, except in subjects with serum ferritin levels > 1000 µg/L. Previously, we showed substantially lower cut-off values for APRI and Fib4 for detection of AHF in HH compared to other liver diseases^17^. We now extend this to demonstrate that Hepascore and TE cut-offs for the detection of AHF in HH are also substantially lower compared to other liver diseases^18–27^. The lower cut-offs probably reflect the lesser degree of hepatic inflammation which occurs in HFE Hemochromatosis liver injury compared with other liver diseases^8,31^.

As all individuals underwent phlebotomy treatment following recruitment into the study, we were able to evaluate the responses to treatment of APRI, Fib4, Hepascore and TE. All subjects, irrespective of the presence or absence of AHF, demonstrated significant reductions in serum ferritin levels with phlebotomy treatment, as expected. Subjects with AHF were older and had higher ALT and/or AST values than those who did not have AHF, compatible with previous reports of HH subjects with liver biopsy-confirmed AHF^2,5,8,17,32^. Furthermore, subjects with nAHF defined on the basis of elevated APRI demonstrated a significant reduction in APRI with phlebotomy treatment whilst those defined on the basis of elevated Fib4 demonstrated significant reductions in APRI and Fib4 with treatment. There were no statistically significant phlebotomy treatment-related effects on Hepascore and TE in subjects with or without nAHF. Our observations are consistent with previous studies which have shown significant reductions in APRI following phlebotomy treatment of subjects with liver biopsy confirmed AHF^17^.

The majority of individuals with HH in our study did not have evidence of AHF and most had serum ferritin levels < 1000 µg/L. Using APRI, Fib4 or the combination of APRI and Fib4, we observed a likelihood of AHF in 13%, 30% or 10% of HH subjects, respectively. Previous population-based studies have reported similar prevalences of between 10 and 25% for AHF in subjects at the time of diagnosis, and primarily in those with serum ferritin levels > 1000 µg/L at the time of diagnosis^2,5,7,9,32^. Interestingly, both Hepascore and TE identified all HH subjects who had serum ferritin levels > 1000 µg/L and who had elevation of APRI above 0.44. Hepascore and TE identified 6 of 8 HH subjects who had serum ferritin levels > 1000 µg/L and who had elevation of Fib4 above 1.1. Thus, Hepascore and TE may be more reliable in the subgroup of individuals who have serum ferritin levels above 1000 µg/L.

Our study has several strengths and weaknesses. While the relatively small sample size could be considered a potential limitation, the strengths of the study include the prospective enrolment and collection of data from community-dwelling subjects who were able to be followed-up after phlebotomy treatment. Subjects enrolled in our study were not required to undergo liver biopsy for routine clinical care and thus we were unable to compare our noninvasive biomarkers with liver-biopsy confirmed fibrosis. However, we believe our use of APRI and Fib4 cut-off values which have been recently validated against liver biopsy-staged fibrosis in HH^17^ is a pragmatic alternative given the relative rarity in routine clinical practice of liver biopsy for evaluation of HH.

## Conclusion

The use of Hepascore or TE for detection of AHF in HH may lead to underdiagnosis, except when individuals have serum ferritin levels elevated above 1000 µg/L. Overall, APRI or Fib4 are more clinically useful than Hepascore or TE for detection of AHF.

## Data Availability

The datasets generated during and/or analysed during the current study are available from the corresponding author on reasonable request.
